# The behavior of larval zebrafish reveals stressor-mediated anorexia during early vertebrate development

**DOI:** 10.3389/fnbeh.2014.00367

**Published:** 2014-10-20

**Authors:** Rodrigo J. De Marco, Antonia H. Groneberg, Chen-Min Yeh, Mario Treviño, Soojin Ryu

**Affiliations:** ^1^Developmental Genetics of the Nervous System, Max Planck Institute for Medical ResearchHeidelberg, Germany; ^2^Laboratorio de Plasticidad Cortical y Aprendizaje Perceptual, Instituto de Neurociencias, Universidad de GuadalajaraGuadalajara, México

**Keywords:** stress response, HPA-axis, feeding, larval zebrafish, behavior

## Abstract

The relationship between stress and food consumption has been well documented in adults but less so in developing vertebrates. Here we demonstrate that an encounter with a stressor can suppress food consumption in larval zebrafish. Furthermore, we provide indication that food intake suppression cannot be accounted for by changes in locomotion, oxygen consumption and visual responses, as they remain unaffected after exposure to a potent stressor. We also show that feeding reoccurs when basal levels of cortisol (stress hormone in humans and teleosts) are re-established. The results present evidence that the onset of stress can switch off the drive for feeding very early in vertebrate development, and add a novel endpoint for analyses of metabolic and behavioral disorders in an organism suitable for high-throughput genetics and non-invasive brain imaging.

## Introduction

Organisms respond to threats by activating a set of processes collectively referred to as the stress response (Selye, 1956; Chrousos, 1998). These processes aim to preserve body homeostasis and rely heavily on the hypothalamic-pituitary-adrenal (HPA) axis (Charmandari et al., 2005). The human hypothalamus contains groups of neurons concerned with the regulation of energy balance, sexual drive and sleep. It responds quickly to a state of threatened homeostasis, or stress, by triggering the release of key hormones from the pituitary, such as adrenocorticotropic hormone (ACTH; Sapolsky, 2000). The adrenal gland (interrenal gland in teleosts) then secretes cortisol under the control of ACTH, released from the pituitary in response to signals from the hypothalamus, such as corticotropin-releasing-hormone (CRH). Thus, external stimuli triggering the release of CRH, ACTH and cortisol are termed stressors, and can exert various effects on behavior. Under non-stressful conditions, for example, food intake and energy expenditure are kept balanced through a process called energy homeostasis (Kennedy, 1953), controled by neurons in the hypothalamus and other brain areas receptive to peripheral metabolic signals (Gao and Horvath, 2007; Morton et al., 2014). Once the HPA-axis has been activated, however, a state of reduced appetite suppresses the normal regulation of energy homeostasis and prevents feeding until stress has ceased (Morton et al., 2014). This phenomenon involves actions by neurons in the hypothalamus and other brain areas (Morton et al., 2014), including the parabrachial nucleus (Carter et al., 2013), and a number of stress mediators, including glucocorticoids (Spina et al., 1996; Carr, 2002; Maniam and Morris, 2012; Matsuda et al., 2013). So far, evidence linking acute stress to reduced appetite has been documented in adults (Carr, 2002), but less so in developing vertebrates. Here, we searched for direct evidence of stressor-mediated anorexia in larval zebrafish (*Danio rerio*).

Because of the ease of handling and their physiological and neuroanatomical homology to humans, zebrafish are becoming increasingly popular in biomedical research and models for behavioral and metabolic disorders are being established (Nguyen et al., 2013). In particular, larval zebrafish appear highly suitable for addressing mechanisms governing stress-induced anorexia. The zebrafish hypothalamic-pituitary-interrenal (HPI) axis shares conspicuous homologies with the HPA-axis in humans (Wendelaar Bonga, 1997; Löhr and Hammerschmidt, 2011), and the larvae’s neurosecretory preoptic-hypothalamic area is comparable to the hypothalamic paraventricular nucleus in mammals (Herget et al., 2014). Also, measures of hormone levels and gene expression analysis show that the stress response can be triggered around 4 days post fertilization (dpf; Alsop and Vijayan, 2008, 2009a,b; Alderman and Bernier, 2009; Steenbergen et al., 2011; Yeh et al., 2013). Further, the energy reservoir (yolk sack) of embryos begins to deplete around 3 dpf (Kimmel et al., 1995), and, by 6 dpf, larvae execute elaborate prey capture behaviors (Borla et al., 2002; McElligott and O’malley, 2005; Trivedi and Bollmann, 2013). Also importantly, due to their small size, genetic access and transparent body, larval zebrafish are suitable for high-throughput behavioral genetics, non-invasive brain imaging and optogenetic probing of neural circuits (Gahtan and Baier, 2004; Portugues et al., 2013). Altogether, these features favour the view that zebrafish larvae may contribute significantly to the study of how stress influences appetite and food consumption, although whether they are subject to stress-induced anorexia remains still unresolved.

Here we show that first encounters with distinct stressors can suppress feeding in larval zebrafish. Using computer-vision-based methods, we first developed a protocol for assessing food consumption in groups of freely swimming larvae. For this, we quantified the larvae’s use of space in an environment with unevenly distributed prey, and validated our measure as an accurate assessment of food consumption. Next, we presented larvae with a well-established, albeit artificial stressor (i.e., hyperosmotic medium) and determined the relationship between ensuing whole-body cortisol and the larvae’s feeding performance. We observed a robust stressor-mediated suppression of feeding. We then examined if such form of food intake suppression could be accounted for by altered locomotion, oxygen consumption or visual reactions. The results showed that it could not, favouring the view that the onset of stress acted on the larvae’s drive for feeding. Finally, in order to ponder the link between stressor identity and stressor-mediated food intake suppression, we also tested feeding in larvae pre-exposed to a novel stress protocol based exclusively on water motions likely to occur in the zebrafish native environment, such as those originating from hovering fish or approaching predators. The results of these tests confirmed that initial stress and reduced feeding appear linked to each other. Also importantly, we found that feeding in stressed larvae reoccurred when basal cortisol levels were re-established. In sum, we found that larval zebrafish display a robust form of stressor-mediated anorexia and introduced a novel endpoint for analyses of stress effects on metabolic and behavioral disorders.

## Material and methods

### Zebrafish husbandry

Zebrafish breeding and maintenance was performed under standard conditions (Westerfield, 2000). Embryos were collected in the morning and raised on a 12:12 light/dark cycle in E2 medium (Westerfield, 2000). All experiments were carried out with wild-type (cross between AB and TL strains) 6 dpf larvae. Zebrafish experimental procedures were performed according to the guidelines of the German animal welfare law and approved by the local government.

### Salt stress

Larvae were incubated for 10 min in steady state E2, E2 + 50 mM NaCl (NaCl_50mM_) or E2 + 100 mM NaCl (NaCl_100mM_) media, at 28°C under white light illumination. They were then washed three times with E2 medium and kept in a small container for oxygen consumption measurements (6–8 min later), or transferred to a custom made swimming chamber for behavioral testing. The wash and transfer period took 3 min (± 10 s) and was performed at room temperature. Behavioral recordings began 5 min after stressor exposure. Cortisol detection was carried out using a home-made cortisol ELISA protocol, as described in (Yeh et al., 2013).

### Mechanosensory stress

Larvae were presented with fast hydrodynamic flows caused by the rapid lateral displacements (LDs) of a partially submerged inflexible silica capillary tube (Polymicro Technologies, AZ, 360 µm OD, Optronis GmbH; Kehl, Germany) fixed to a multilayer piezo bender actuator (PICMA® PL140.10, Physik Instrumente (PI) GmbH and Co. KG; Karlsruhe, Germany). The actuator had an operating voltage of 0–60V, a maximum displacement of ±1000 µm, and an unloaded resonant frequency of 160 Hz. The bender was connected to a dual-piezo-amplifier (maximum voltage: 10 V), a pulse generator and a TTL control system allowing for computer control. The tip of the silica capillary tube was placed, partially submerged (2 mm), at the center of a 35 mm petri dish half filled (1.8 ml) with E2 medium (orientation relative to water surface: 90°). The voltage applied to the bender (V_act_) determined the speed of the capillary’s LDs, or stimulus strength (in % relative to maximum voltage). Groups of 30 larvae were exposed to 6 stimulation units delivered with an inter-stimulation-interval of 250 ms. Each unit consisted of 99 40 ms LDs. We used a V_act_ of 3, 4.5 or 6 V. Stimulations were carried out under white illumination at 25°C. After stimulation, larvae were kept in the petri dishes for cortisol measurement (9.5 min later) as described in (Yeh et al., 2013), or transferred to a custom made swimming chamber for behavioral testing, where they remained unperturbed for 10 min before recordings.

### Oxygen consumption

Oxygen levels inside a single custom made flow chamber with 8 freely swimming larvae were monitored (as % air saturated) every 5 s for 30 min using fibre optic sensing technology (PreSens Precision Sensing GmbH; Regensburg, Germany). To minimize perturbations during recordings, the chamber was placed inside a light-proof box. Its inlet and outlet channels had silicon tubes (750 µm OD) and a 5 ml Luer-Lok™ Tip Syringe (BD, Heidelberg, Germany) was connected to the inlet to fill the chamber with solution; both inlet and outlet remained closed during measurements. To calibrate the oxygen-sensitive sensor, we used oxygen-free (Cal 0) and air-saturated water (Cal 100). For Cal 0, Na_2_SO_3_ (0.2 g) was dissolved in distilled water (20 ml). For Cal 100, air was blown in distilled water (100 ml) for 20 min while stirring; air flow was then stopped and the solution stirred for another 10 min to avoid hyper-saturation. We used a two-point calibration with temperature and atmospheric pressure compensation. For each measurement, the oxygen consumption rate (OCR) was approximated as the slope of a linear fit to the oxygen level for 10 min < time < 30 min. Prior to measuring the OCRs of larvae, the OCR of E2 alone was measured twice and averaged. Net OCRs of the larvae were calculated by subtracting the average OCR of E2 of the actual day from the OCRs of the larvae.

### Behavioral testing

Experiments on feeding were conducted under both white and infrared (IR) light, delivered through a custom-made array of white- and IR-LEDs mounted inside a custom-made light-proof enclosure placed on a vibration-free platform (Newport, Irvine, CA, USA). Avoidance and individual locomotion tests were conducted under IR light only. In all cases, larvae were imaged at 25 frames s^−1^ through an infrared-sensitive camera (ICD-49E B/W; Ikegami Tsushinki Co., Ltd. Japan) with its lens (TV Lens, Computer VARI FOCAL H3Z4512 CS-IR, CBC; Commak, NY, USA) positioned above a swimming chamber. We used EthoVision XT software (Noldus Information Technology; Wageningen, The Netherlands) and algorithms written in MATLAB 7.8 (MathWorks, Inc.; Natick, MA, USA) to monitor the movements of larvae swimming either individually (avoidance and individual locomotion tests) or in groups (feeding) inside custom-made chambers, respectively. In all experiments, larvae were allowed to adjust to the chamber conditions for several minutes prior to recording. Experiments were conducted at 28 ± 1°C. A thermocouple (npi electronics GmbH; Tamm, Germany) connected to a temperature control system (PTC 20, npi electronics GmbH; Tamm, Germany; Exos-2 V2 liquid cooling system, Koolance; Auburn, WA, USA) monitored the temperature inside the chambers. All the experiments were performed in a blind fashion as to group identity. Control animals for each group were handled in the same fashion, but omitting stimulus presentation. Tests were conducted between 9:00 and 18:00 and different experimental groups intermixed throughout the day.

#### Feeding test

Groups of twenty larvae pre-exposed to either E2 medium alone or salt or mechanosensory stress were placed in a custom-made swimming chamber (length: 40 mm, width: 20 mm, height: 10 mm). Two vertically-oriented transparent walls (width: 500 µm) divided the chamber into three contiguous compartments, S1, Centre and S2, with the Centre compartment being twice as large as each of the side compartments. The transparent walls divided only the upper part (eight tenth) of the cross section of the chamber, leaving space at the bottom (2 mm) for the larvae to move across compartments while a camera placed above recorded their movements under white light illumination. The walls also had contiguous rows of equidistant 100 µm openings allowing the medium to diffuse. Also, they acted as barriers to confine paramecia to a single compartment, either S1 or S2. Their size, slow motion and tendency to remain in the upper part of the water column prevented paramecia from moving from one compartment to another during the 600 s of each recording session. Following acclimation, video-recordings were made first in the absence of food items (initial 10 min) and, then, after having added a known quantity of paramecia to one of the chamber’s side compartments, either S1 or S2 (final 10 min). When assessing food consumption, paramecia were counted before and after the final 10 min video-recordings. The resulting differential space use (DSU) values were calculated and compared across groups.

#### Quantifying motion and space use

To measure motion and space use, we used an algorithm written in MATLAB 2009b (MathWorks, Inc.; Natick, MA, USA) which detects the movements of larvae swimming within one or several areas of interest using the pixel-by-pixel mean squared error (*m.s.e*) of gray-scale transformed and adjusted images from consecutive video-frames, given by:

(1) $$m.s.e.=\frac{1}{N}\sum_{pixel=1}^{N}{\left(image_{frame,pixel}-image_{frame-1,pixel}\right)}^{2}$$

where N corresponds to the total number of pixels of each frame. We confirmed that *m.s.e.* remained constant in empty chambers, and that *m.s.e.* changes were exclusively due to the movements of swimming larvae. Also importantly, in our experiments simultaneous *m.s.e.* data from multiple areas (e.g., compartments) could be parsed and *m.s.e.* remained insensitive to moving paramecia due to the limited spatial resolution of the camera. Larval zebrafish are highly sensitive to photic stimuli (Burgess and Granato, 2007a), and react to sharp transitions from darkness to light with stereotypic changes in locomotion (MacPhail et al., 2009). Measurements from experiments where single or groups of dark adapted larvae were exposed to consecutive squared pulses of light of varying length and strength validated the *m.s.e.* index as a highly sensitive motion detector. The *m.s.e.* index is also a derivative of the number of larvae swimming within a given area of interest. Independent measurements from experiments varying the number of larvae (min: 1, max: 10) swimming within a small cylindrical chamber (10 mm ID) showed that, even over a time period as short as 10 s, the relationship between *m.s.e.* and the number of larvae could be well approximated by a linear regression (*p* < 0.0001, R-squared: 0.98), indicating that the *m.s.e.* index is sensitive to both the amount of motion and the number of swimming larvae.

#### Reaction to illumination change

Larvae pre-exposed to either E2 medium alone or a hyperosmotic medium (NaCl_50mM_ or NaCl_100mM_) were placed individually in a custom-made rectangular chamber (length: 40 mm, width: 20 mm, height: 10 mm) kept in darkness and filled with 3 ml of E2 medium. Following a 10 min acclimation period, single larvae were imaged at 25 frames s^−1^ for 120 s. Each video-recording consisted of an initial period of 60 s under IR illumination, followed by a 10 s square pulse of white light (0.1 mW*cm^−2^) and a final 50 s period under IR illumination. EthoVision XT software (Noldus Information Technology; Wageningen, The Netherlands) was used to monitor the movements of the single larvae; group comparisons were based on their average swim velocity (calculated over 10 s) before, during and after light exposure.

#### Optomotor response

Larvae pre-incubated in either E2 medium or a hyperosmotic medium (NaCl_50mM_ or NaCl_100mM_) were placed individually in a rectangular chamber (length: 40 mm, width: 20 mm, height: 10 mm) filled with 3 ml of E2 medium and mounted directly above a computer screen horizontally oriented. The screen displayed visual stimuli created via an algorithm written in MATLAB 2009b (MathWorks, Inc.; Natick, MA, USA). Visual stimuli consisted of gray dots (displayed against a white background offering a light power of 0.5 mW*cm^−2^) of variable contrast (%), diameter (in degrees), velocity (in degrees s^−1^) and number, as defined at a viewing distance of 2 mm. We used EthoVision XT software (Noldus Information Technology; Wageningen, The Netherlands) to monitor the movements of larvae swimming individually at 28 ± 5°C. In each recording session, single larvae were first presented with stationary dots and allowed to swim freely for 60 s. Next, as soon as they reach any of the chamber’s sides (either left or right), the dots began to move towards the opposite side of the chamber. Under these circumstances, larvae would align themselves with the moving dots and begin to swim consistently towards the opposite side of the chamber. To quantify their optomotor response, we measured the time interval in between when they started to move together with the dots and when they reached the opposite side of the chamber (latency, in seconds). The effects of dot contrast, diameter, velocity and number were tested by means of independent recordings.

### Statistical analysis

All data are shown as single measurement points, mean and standard error of the mean (S.E.M.) or box-and-whisker plots. We used paired Student’s *t*-tests (two-tailed) for two-group comparisons as well as ANOVAs, followed by Bonferroni’s *post-hoc* tests, or Kruskal-wallis tests (if the data did not fulfill the assumptions of the ANOVA), followed by Dunn’s tests, for multiple group comparisons. We also used linear and non-linear regression analysis. Analyses were carried out using MS-Excel (Microsoft; Redmond, WA, USA), MATLAB 2009b (MathWorks, Inc.; Natick, MA, USA), Prism 5, (Graphpad Software Inc.; San Diego, CA, USA), Sigma Plot (Systat Software Inc.; San Jose, CA, USA), R (Freeware) and Virtual Dub (Freeware).

## Results

### Measuring food consumption in groups of freely swimming larvae

**Figure 1:**
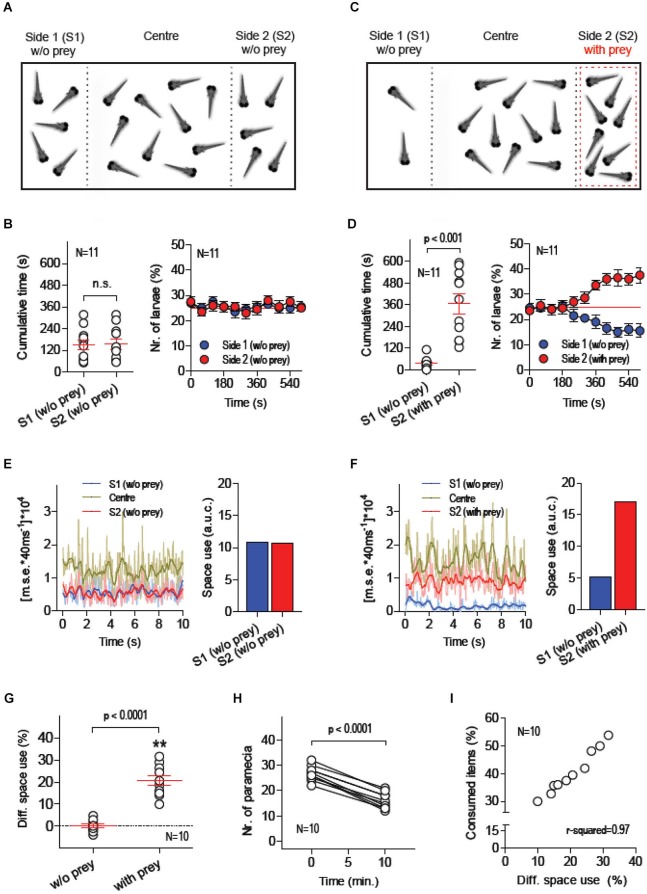
**Measuring food consumption in groups of freely swimming larvae**. **(A–C)** Schematic of the basic experimental procedure: 20 freely behaving larvae are placed in a custom-made swimming chamber composed of three interconnected compartments (S1, Centre and S2). A camera placed above monitors their movements under white light illumination during two consecutive 10 min periods, first in the absence of food items (**A**, initial 10 min) and then in the presence of a defined quantity of paramecia (prey) in one of the chamber’s side compartments, either S1 or S2 (**C**, final 10 min, prey in S2). The position of each larva and the overall activity of the group in S1, Centre and S2 are recorded. **(B,D)** Time spent by each larva and average percentage of larvae in S1 and S2 in the absence **(B)** and presence **(D)** of prey. **(E,F)** Exemplary 10 s traces of [mean squared error*40 ms^−1^]*10^4^ (a measure of global motion matching the number of swimming larvae) for each of the chamber’s compartments (left), and resulting ‘space use’ (right, i.e., area under the curve from S1- and S2-traces) in the absence **(E)** and presence **(F)** of prey (see Methods). **(G)** Differential space use (DSU, in %), determined as “space use” in S2 relative to that in S1, with and without prey (asterisks designate results from a one-sample *t*-test against 0, *p* < 0.01). **(H)** Number of paramecia in “side with prey” (either S1 or S2) at the beginning and the end of the final 10 min recording period. **(I)** DSU variance accounts for 97% of the variance of consumed paramecia (in %, relative to the total amount of paramecia at the beginning of the 10 min session).

Twenty larvae were placed in a custom made swimming chamber where two transparent walls created three contiguous compartments: S1, Centre and S2 (Figure 1A). The Centre compartment, twice as large as the side compartments, acted as a transition zone between S1 and S2. The transparent walls divided only the upper part of the chamber’s cross section, leaving space at the bottom for the larvae to move freely across compartments while a camera placed above recorded their movements under white light illumination. The walls contained rows of small openings allowing the medium to diffuse and acted as barriers to confine moving prey (paramecia), or food items, to a single side compartment, either S1 or S2 (see Methods). Each recording session lasted 600 s. Larvae were allowed 10 min of acclimation before recordings started. Following acclimation, video-recordings were made first in the absence of food items (initial 600 s) and, then, after having added a known quantity of paramecia to one of the chamber’s side compartments, either S1 or S2 (final 600 s). In the absence of prey and external stimuli (other than visual and hydrodynamic cues caused by their own movements), larvae spent equal amounts of time in S1 and S2 (Figure 1B, left: Paired *t*-test, *t*_(10)_ = 0.1, *p* = 0.92). On average, the percentage of larvae found in either S1 or S2 was ~25%, as expected from a symmetric distribution of individuals within the chamber (Figure 1B, right). This changed when paramecia were added to one of the side compartments, either S1 or S2 (Figure 1C). The presence of prey reliably triggered a differential space use (DSU) by the larvae. They spent more time in the compartment with paramecia (Figure 1D, left: Paired *t*-test, *t*_(10)_ = 5.8, *p* = 0.0002), increasing the number of individuals in it after 600 s (Figure 1D, right). To quantify the overall motion and the number of larvae swimming in specific areas of the chamber, we used a tracking algorithm which computes the pixel-by-pixel *m.s.e.* between images from consecutive video-frames (Figures 1E,F, see also Methods). First, we calculated the area under the curve (a.u.c.) from ensued *m.s.e.* values (in 600 s) in order to estimate the amount of larvae that swam concurrently within each of the chamber’s compartments. Using these values, we then calculated a global measure of DSU, as the difference (in %) between the a.u.c. values from S1 and S2, or [(S1_a.u.c._ – S2_ a.u.c._)/(S1_ a.u.c._ + S2_a.u.c._)]*100, always using data from the side with prey as S1. A comparison of DSU values recorded with and without paramecia validated the approach as an accurate method for quantifying the relation between the spatial distributions of both prey and larvae (Figure 1G, Paired *t*-test, *t*_(9)_ = 10.8, *p* < 0.0001). Importantly, by counting the number of paramecia consumed by the group of larvae (Figure 1H, Paired *t*-test, *t*_(9)_ = 17.2, *p* < 0.0001), we observed that DSU variability accounted for 97% of the variance of prey consumption (Figure 1H, Pearson’s correlation, *p* < 0.0001). Further, a comparison of total a.u.c. values (i.e., summed values from S1, Centre and S2) showed that prey-dependent DSU changes could not be accounted for by changes in the overall level of motion displayed by larvae throughout the recording periods (Paired *t*-test, *t*_(9)_ = 0.21, *p* = 0.84).

### Exposure to a hyperosmotic medium triggers HPI-axis activation

**Figure 2:**
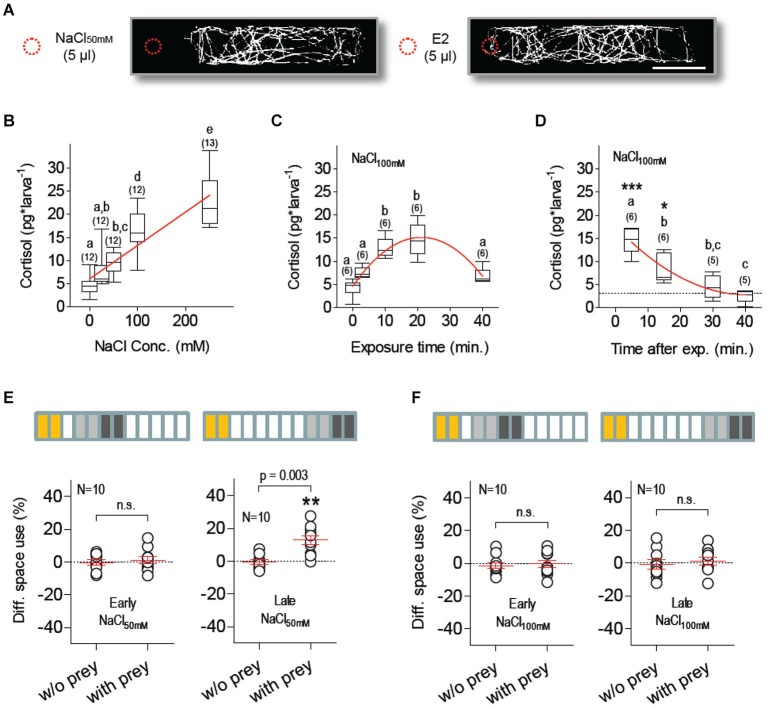
**Salt stress suppresses feeding**. **(A)** Exemplary tracks from larvae swimming in darkness at constant temperature (scale bar: 10 mm). Traces from single larvae appear biasedly distributed when 5 µl of NaCl_50mM_ (left), but not of E2 (right), are added (dashed red circle) to the 3 ml E2 medium in the chamber, revealing active avoidance of sharp salinity variations. **(B)** NaCl exposure increases whole-body cortisol in a dose-dependent manner; linear regression (*p* < 0.0001) designated by red line. **(C)** Maximum cortisol levels are detected 10–20 min after NaCl exposure; as exposure length increases, cortisol drops after reaching a peak; non-linear regression (R-square *=* 0.78) designated by red line. **(D)** Stress-induced cortisol reach basal levels 30 min after NaCl exposure (dashed line denotes average basal levels); non-linear regression (R-square *=* 0.78) designated by red line. (**B–D**: different letters designate statistical differences determined by one-way ANOVAs followed by *post-hoc* comparisons. Sample size in parentheses). **(E,F)** DSU in larvae pre-incubated with either NaCl_50mM_ **(E)** or NaCl_100mM_ **(F)**. Top bars: each rectangle represents a 5 min time period; from left: NaCl exposure (yellow), first 10 min period without prey (light gray), second 10 min period with prey (dark gray). Shown are DSU values measured either 5–25 min (left, early) or 30–50 min (right, late) after NaCl exposure. Asterisks designate results from a one-sample *t*-test against 0, *p* < 0.01.

Salt exposure is a well-established stressor in teleosts and has been shown to increase whole-body cortisol in larval zebrafish (Yeh et al., 2013). We observed that freely swimming larvae avoided a sudden increase of salt concentration, as they rapidly moved away from a spot where 5 µl of 50 mM NaCl solution was added to their medium and continued to avoid the vicinity of the addition spot over a 180 s video-recording period; such a series of avoidance reactions did not occur when 5 µl of E2 were added to the medium (Figure 2A). In separate experiments, we next measured whole-body cortisol as a function of (1) NaCl concentration in a steady-state hyperosmotic medium, (2) NaCl exposure duration; and (3) time after exposure (Figures 2B–D). The results showed that NaCl exposure increased cortisol in a concentration-dependent manner (Figure 2B, Kruskal-Wallis test, *H* = 48.6, *p* < 0.0001, followed by Dunn’s multiple comparison tests and linear regression), that maximum cortisol levels were detectable 5–10 min after NaCl exposure (Figure 2C, One-Way ANOVA, *F*_(4,29)_ = 22.8, *p* < 0.0001, followed by Bonferroni post-tests for pair comparisons and non-linear regression), and that basal levels were re-established 30 min later (Figure 2D, One-Way ANOVA, *F*_(3,23)_ = 25.4, *p* < 0.0001, followed by Bonferroni post-tests for pair comparisons and non-linear regression).

### Salt stress suppresses feeding

To investigate how HPI-axis activation influences feeding in freely swimming larvae, we measured prey-dependent DSU changes in larvae that had been previously exposed (for 10 min) to hyperosmotic E2 media containing additional NaCl (NaCl_50mM_ or NaCl_100mM_). Both incubations (NaCl_50mM_ and NaCl_100mM_) suppressed prey-dependent DSU changes 15–25 min after the offset of NaCl exposure (Figures 2E,F, left, Paired *t*-tests, NaCl_50mM_: *t*_(9)_ = 0.51, *p* = 0.62, NaCl_100mM_: *t*_(9)_ = 0.38, *p* = 0.72). Later, 40–50 min after exposure, prey-dependent DSU changes were detectable in larvae pre-incubated with NaCl_50mM_ but not with NaCl_100mM_ (Figures 2E,F, right, Paired *t*-tests, NaCl_50mM_: *t*_(9)_ = 4.02, *p* = 0.003, NaCl_100mM_: *t*_(9)_ = 0.51, *p* = 0.63). Larvae pre-incubated with NaCl_100mM_ showed normal prey-dependent DSU changes, i.e., indistinguishable from those of control larvae, three hours after NaCl exposure (Paired *t*-test, *t*_(9)_ = 4.3, *p* = 0.002). Control larvae (pre-incubated with normal E2 medium) showed prey-dependent DSU changes 15–25, 40–50 and 180–190 min after incubation (Paired *t*-tests, 15–25 min: *t*_(9)_ = 8.72, *p* < 0.0001, 40–50 min: *t*_(9)_ = 7.1, *p* < 0.0001, 180–190 min: *t*_(9)_ = 4.6, *p* = 0.001). A comparison of the total a.u.c. values from all groups indicated that motion variability could not account for the observed prey-dependent changes in DSU (Two-Way Repeated-Measures ANOVA, Group factor: *F*_(7,72)_ = 0.56, *p* = 0.78, Prey factor: *F*_(1,72)_ = 0.21, *p* = 0.65, Group X Prey factor: *F*_(7,72)_ = 0.12, *p* = 0.99).

### Locomotion, oxygen consumption and visual responses remain unaltered after stressor exposure

**Figure 3:**
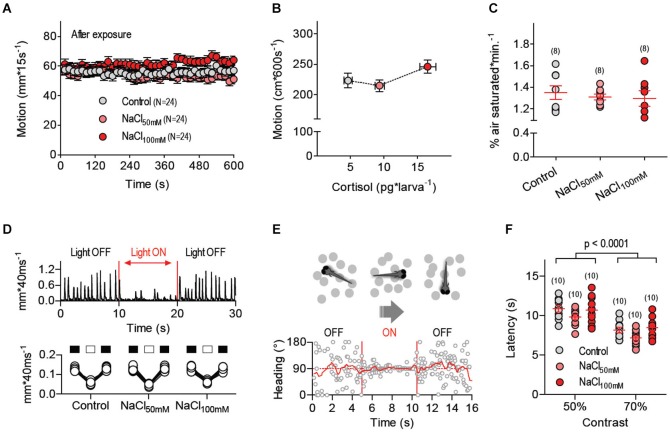
**Locomotion, oxygen consumption and visual responses remain unaltered after stressor stress. (A)** Locomotor activity of larvae pre-incubated with E2, NaCl_50mM_ and NaCl_100mM_ swimming individually in darkness at 28°C (±0.1°C). **(B)** Average locomotion (over 600 s) plotted against whole body cortisol for larvae pre-treated with E2, NaCl_50mM_ and NaCl_100mM_. **(C)** Net oxygen consumption rates (OCRs) of groups of (eight) larvae pre-treated with E2, NaCl_50mM_ or NaCl_100mM_. (**A,C**: sample size in parentheses). **(D)** Swim velocity of single dark-adapted larvae pre-treated with E2, NaCl_50mM_ or NaCl_100mM_ before, during and after a 10 s square pulse of white light (0.1 mW*cm^−2^). **(E)** Exemplary trace from an optomotor test depicting a larva’s heading (relative to the long axis of a rectangular swimming chamber) as a function of time; during the test, single larvae are presented with gray dots (of variable contrast, diameter, velocity and number) ventrally displayed against a white background via a computer screen beneath the chamber (see Methods). No preferred heading can be detected when the dots remain stationary (OFF); by contrast, larvae swim in the direction of the moving dots when they move along the long axis of the chamber (ON), thereby showing a preferred heading. Following acclimation and baseline recording, dot movements begin automatically when the larva is at one of chamber’s sides (either left or right); dots move towards the chamber’s opposite side and the time elapsed until the larva reaches the end of the chamber (latency) is taken as indicative of response strength. **(F)** Latency (s) from an optomotor test as a function of dot contrast for larvae pre-treated with E2, NaCl_50mM_ or NaCl_100mM_.

We next asked whether altered locomotion, oxygen consumption or visual reactions could account for the differences in prey-dependent space use observed after NaCl exposure. We first monitored post-exposure levels of locomotor activity in treated and control larvae within a stimulus-deprived environment. For this, larvae pre-incubated in either control or hyperosmotic mediums were placed in a custom-made, vibration-free swimming chamber (10 mm ID, volume: 400 µl) and allowed to swim individually in complete darkness for 600 s under constant temperature (28 ± 0.1°C). We found that NaCl exposure failed to modify post-exposure locomotion (Figure 3A, One-Way ANOVA, *F*_(2,71)_ = 2.2, *p* = 0.13); as a result, peak cortisol levels after salt exposure did not account for locomotion variability (Figure 3B, Pearson’s correlation, *p* = 0.42). To expand the analysis of NaCl effects, we monitored oxygen levels every 5 s for 30 min (as % of air saturated) in a custom-made chamber containing groups of eight freely swimming larvae that had, or had not, been pre-incubated in a hyperosmotic medium (of either NaCl_50mM_ or NaCl_100mM_). For each measurement, the OCR was approximated as the slope of a linear fit to the oxygen level for 10 min < time < 30 min. Prior to measuring the OCRs of larvae, we measured (twice) the OCR of E2 medium alone. Net OCRs were calculated by subtracting the average OCR of E2 from the OCRs of the larvae. We found that pre-incubation with either NaCl_50mM_ or NaCl_100mM_ did not change net OCRs, as compared to pre-incubation with E2 (Figure 3C, One-Way ANOVA, *F*_(2,23)_ = 0.26, *p* = 0.77). Next, because larvae rely on vision for detecting and capturing prey, we set up to detect possible effects of NaCl exposure on their reactions to illumination change and moving visual stimuli. First, we examined motion reactions of control and treated larvae to a sudden illumination change and observed that dark-adapted larvae of either group reacted similarly to a 10 s square pulse of white light (0.1 mW*cm^−2^). They reduced their locomotor activity similarly when exposed to light (Figure 3D, Two-Way Repeated-Measures ANOVA, Group factor: *F*_(1,36)_ = 0.41, *p* = 0.53, Light factor: *F*_(2,36)_ = 391.7, *p* < 0.0001, Group X Light factor: *F*_(2,36)_ = 0.69, *p* = 0.51). Next, we tested the larvae’s optomotor response, i.e., spontaneous swimming in the direction of large-field displacements in the visual field. For this we presented control and treated larvae with ventrally moving dots of variable contrast, diameter, velocity and number (Figure 3E, see Methods). The results showed that NaCl exposure did not impair the larvae’s optomotor response. Figure 3F shows how dot contrast improved the larvae’s response, and how the response of larvae pre-incubated in either NaCl_50mM_ or NaCl_100mM_ did not differ from that of control larvae (Two-Way ANOVA, Group factor: *F*_(2,54)_ = 2.49, *p* = 0.09, Contrast factor: *F*_(1,54)_ = 69.57, *p* < 0.0001, Group X Contrast factor: *F*_(2,54)_ = 0.59, *p* = 0.56). Similar results arose from varying the diameter, velocity and number of moving dots (data not shown). In sum, locomotion, oxygen consumption and responses to visual inputs appeared unaltered under conditions of elevated HPI-axis activity caused by exposure to hyperosmotic mediums.

### Mechanosensory stress suppresses feeding

**Figure 4:**
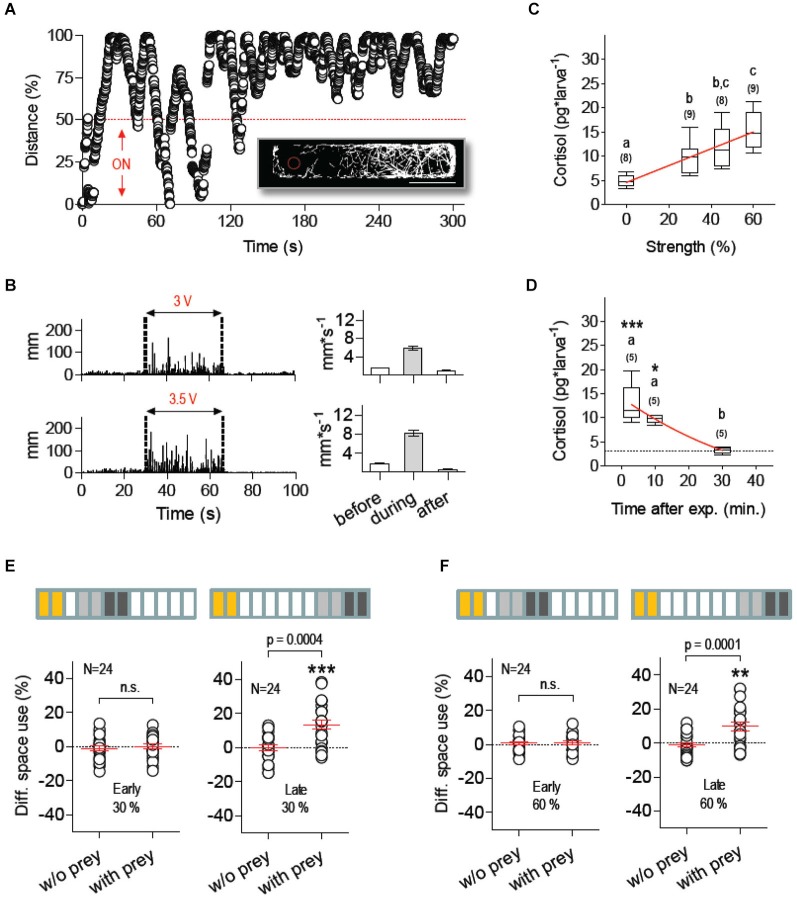
**Mechanosensory stress suppresses feeding**. **(A)** Distance (in % relative to maximum) every 40 ms between a single larva swimming in darkness at 28°C (±0.1°C) and the submerged tip of a silica capillary tube fixed to a computer-controled piezo bender actuator. The capillary’s tip (stimulus source) moves laterally upon voltage applied to the actuator, thereby causing fast hydrodynamic flows. The bender’s LDs (of known frequency and duration) can be triggered at any given time or only when the larva swims within pre-defined areas of the swimming chamber, such as the half of the chamber containing the stimulus source (ON, bottom). Larvae respond to the stimulus by increasing the distance to the source. Insert: x-y coordinates from an exemplary 300 s track illustrating how a single larva avoids the area surrounding the source (red dashed circle, scale bar, 10 mm). **(B)** Left: distance swam by representative larvae before, during (between dashed lines) and after stimulation of increasing stimulus strength (delivered at 1 Hz), as determined by the voltage applied to the bender (V_act_), either 3 (top) or 3.5 V (bottom); right: average distance swam before, during and after stimulation. **(C)** Whole-body cortisol measured 10 min after stimulation as a function of stimulus strength (in % relative to maximum V_act_); linear regression (*p* < 0.0001) designated by red line. **(D)** Cortisol level as a function of time after stimulation (stimulus strength: 60%). Asterisks designate statistical differences as compared to basal levels (*p < 0.05, ***p < 0.001). Non-linear regression (R-square *=* 0.77) designated by red line (**C,D**: different letters designate statistical differences determined by one-way ANOVAs followed by *post-hoc* comparisons; sample size in parentheses). **(E,F)** DSU in larvae pre-exposed to mechanosensory stress using stimulus strength levels of 30% **(E)** and 60% **(F)**, defined as in **(C)** (asterisks designate results from one-sample *t*-tests against 0, **p < 0.01, ***p < 0.001). Top bars: each rectangle represents a 5 min time period; from left: mechanosensory stimulation (yellow), first 10 min period without prey (light gray), second 10 min period with prey (dark gray). Shown are DSU values measured either 5–25 min (left, early) or 30–50 min (right, late) after mechanosensory stimulation.

We next examined the extent to which stressor identity accounted for the effect of NaCl exposure on food intake. We therefore measured prey-dependent DSU changes in larvae pre-exposed to a novel stress protocol based exclusively on mechanosensory stimuli. To evoke mechanosensory stress, we used fast water movements caused by the rapid LDs of an inflexible silica capillary (360 µm OD) submerged partially (2 mm) in the larvae’s surrounding medium. The capillary was fixed to a computer-controled piezo bender actuator and the voltage applied to the actuator (or stimulus strength, in % relative to maximum voltage) determined the speed of the capillary’s LDs (maximum displacement: ±1000 µm). To examine how larvae reacted to the capillary’s LDs, we placed them individually in a rectangular swimming chamber and LDs of known frequency and duration were triggered only when they swam within the half of the chamber containing the tip of the capillary (stimulus source). Larvae responded to LDs by increasing rapidly their distance to the centre point of the moving capillary (Figure 4A). In line with this, their overall locomotion during continuous stimulation (i.e., series of 10 2-ms LDs delivered at 1 Hz, irrespective of the larva’s position relative to the source) increased together with stimulus strength (Figure 4B). We thus assumed that LD-borne stimuli resembled, at least partially, motion and pressure waves (cues) encoding predation threat, such as those derived from the movements of larger fish and approaching predators. Notably, such a form of repeated mechanosensory stimulation increased whole-body cortisol in a stimulus strength-dependent manner (Figure 4C, Kruskal-Wallis test, *H* = 22.4, *p* < 0.0001, followed by Dunn’s multiple comparison tests and linear regression), and post-peak cortisol levels were undistinguishable from basal levels 30 min after stimulation (Figure 4D, Kruskal-Wallis test, *H* = 10.8, *p* = 0.0045, followed by Dunn’s multiple comparison tests and non-linear regression). An analysis of space use after mechanosensory stimulation showed that low (30%) and moderate (60%) stimulus strength levels abolished prey-dependent DSU changes 15–25 min after exposure to LDs (Figures 4E,F, left, Paired *t*-tests, 30%: *t*_(23)_ = 0.48, *p* = 0.63, 60%: *t*_(23)_ = 0.004, *p* = 0.99). Normal DSU changes were detectable 40–50 min later (Figures 4E,F, right, Paired *t*-tests, 30%: *t*_(23)_ = 4.2, *p* = 0.0004, 60%: *t*_(23)_ = 4.6, *p* = 0.0001). As before, across group comparisons of total a.u.c. values indicated that locomotion variability could not account for prey-dependent DSU changes (Two-Way Repeated-Measures ANOVA, Group factor: *F*_(5,54)_ = 0.38, *p* = 0.86, Prey factor: *F*_(1,54)_ = 0.40, *p* = 0.53, Group X Prey factor: *F*_(5,54)_ = 0.20, *p* = 0.96).

## Discussion

The small size, genetic access and transparency of larval zebrafish make them suitable for probing brain elements and peripheral effectors governing processes counteracting stress. For example, optogenetically induced hypercortisolaemia (De Marco et al., 2013) can be used to specify the effect of enhanced pituitary-interrenal responses on stressor-mediated anorexia. However, despite advancements in techniques for altering and measuring specific cell activity (Friedrich et al., 2010), meaningful endpoints reflecting the consequences of stressful events in zebrafish larvae have lagged behind. We show that larval zebrafish execute avoidance reactions to salinity variance and threatening water motions, that exposure to either hyperosmotic mediums or potent hydrodynamic stimuli increases whole-body cortisol in a stimulus strength-dependent manner, and that both such treatments can lead to a full suppression of food consumption. Furthermore, we show that feeding reoccurs when basal cortisol levels are re-established.

If reversible, changes in responsiveness to external stimuli, such as those derived from living prey, are called motivational changes (McFarland, 1971), whereas sets of interacting elements controling groups of related activities (Tinbergen, 1951), such as those serving food search and feeding, are called motivational systems, with system variables defining states of increased or decreased hunger, for example. Understanding the basis of motivational change is a fundamental problem in stress research, where distinct behavioral categories are being influenced by assortments of hormones exerting pleiotropic actions over variable time periods (Morton et al., 2014). Nonetheless, the study of motivational change is challenged by the fact that systemic parameters and state variables can be influenced by a number of physiological processes, triggered, for example, by environmental stimuli, maturation and learning, a fact that introduces non-linear and non-stationary phenomena into the analysis (McFarland, 1971). For example, increased CRH levels can alter locomotion (Lowry and Moore, 2006) and, consequently, also food search activities. Likewise, stress mediators can interact with visuomotor pathways and modify the regulation of prey-capture movements (Carr, 2002). We observed that an encounter with a potent stressor suppressed feeding without causing altered locomotion, oxygen consumption and visual reactions, indicating that the onset of stress can directly alter the larvae’s motivation to feed, thus prompting a state stressor-mediated anorexia. Our observations are in line with the fact that, except upon initial contact with a stressor or under severe conditions of prolonged stress (Selye, 1956; Clark et al., 2011), tightly regulated bodily functions like motor control, energy balance and mechanisms for weighting external stimuli tend not to exceed their normal limits of variation (Selye, 1950; Teichner, 1968). The results are relevant for several reasons.

First, exposure to stressors and stress mediators have often been linked to food intake suppression in adults, but less so in developing vertebrates. The link between stress and reduced food consumption is well documented in adult teleosts, for example. Crowding and handling (Upton and Riley, 2013), as well as cortisol administration (Janzen et al., 2012), have been shown to inhibit food intake in tilapia. Confinement caused elevated cortisol and suppressed feeding in salmon (Pankhurst et al., 2008). Hypoxia (Bernier et al., 2012) and salt exposure (De Boeck et al., 2000) reduced food intake in common carp, and crowding (Conde-Sieira et al., 2010) and cortisol implants (Gregory and Wood, 1999) inhibited food consumption in rainbow trout. Also, social subordination has been found to abolish feeding in arctic char (Øverli et al., 1998), whereas CRH-injection impaired food intake in goldfish (de Pedro et al., 1997). Furthermore, CRF, urotensin I, and serotonin (5-HT) have recently been identified as anorexigenic agents in rainbow trout (Ortega et al., 2013). In adult zebrafish, pre-exposure to either aversive stimuli or alarm cues caused reduced feeding (Oswald and Robison, 2008). Also, just as in humans, where the effect of stress on food intake can be either positive or negative, models of both stress-induced anorexia and stress-induced obesity are being advanced (Merali et al., 2013), highlighting the complexity of the link between stress and metabolic disorders. It has been shown, for example, that adult zebrafish exposed to alarm signals gained weight (as compared to non-exposed individuals) only if they were on a high-fat diet (Nguyen et al., 2013). In developing vertebrates, including teleosts, only a few studies have related adverse conditions and stress hormones to reduced feeding. It has been shown, that CRH administration inhibited feeding in neonatal boiler chicks (Furuse et al., 1997), and that exogenous corticosterone increased the latency to beg for food and suppressed growth in white-crowed sparrow nestlings (Wada and Breuner, 2008). In both pre- and pro-metamorphic tadpoles, intracerebroventricular CRF injection inhibited food intake, and the CRF antagonist alpha-helical CRF suppressed food intake in pro-metamorphic tadpoles (Crespi and Denver, 2004, 2005). Also in tadpoles, altered rearing conditions (i.e., varying population density and food availability) increased corticosteroid levels, which appeared correlated to reduced growth (Glennemeier and Denver, 2002a,b). What has persistently been absent in developing vertebrates is evidence linking stressors to “appetite” (central drive) suppression. The results presented here provide first evidence for stressor-mediated anorexia occurring very early in vertebrate development.

Second, escape reactions to different forms of motion and pressure waves have long been reported in larval zebrafish (Kimmel et al., 1974; Zeddies and Fay, 2005; Burgess and Granato, 2007b; Best et al., 2008; McHenry et al., 2009; Roberts et al., 2011; Buck et al., 2012; Kohashi et al., 2012; Olszewski et al., 2012; Bhandiwad et al., 2013; Stewart et al., 2013). Our experiments introduce a novel stress protocol based on fast water motions resembling motion and pressure waves likely to occur in the zebrafish native environment, such as those caused by larger fish and approaching predators (Engeszer et al., 2007; Spence et al., 2008). Because varying levels of salt, pH, EtOH, heavy metals and severe swirling or vortexing have been shown to increase cortisol in larval zebrafish (Alsop and Vijayan, 2008, 2009a,b; Alderman and Bernier, 2009; Steenbergen et al., 2011; Yeh et al., 2013), the protocol described here will aid effort to compare effects of purely physical and more complex physico-chemical stressors.

Third, the results add a novel assay to the repertoire of laboratory tests of larval zebrafish, an organism increasingly popular in translational research that still lacks sufficient behavioral assessments. Larval zebrafish allow for *in vivo* analyses of brain function and behavior and may offer an excellent opportunity for dissecting short- and long-term effects of stressors on nervous and hormonal control systems. Adding to previous works, our data provide a behavioral endpoint suitable to be combined with pharmacological and genetic targeting of specific cells within the HPI-axis, as well as optogenetic circuit analysis, in order to examine metabolic and behavioral disorders.

## Author contributions

Conception and design of the experiments: Rodrigo J. De Marco and Soojin Ryu. Acquisition of data: Rodrigo J. De Marco, Antonia H. Groneberg, Chen-Min Yeh. Development of MATLAB scripts: Mario Treviño. Analysis and interpretation of data: Rodrigo J. De Marco, Antonia H. Groneberg, Soojin Ryu. Writing the article: Rodrigo J. De Marco and Soojin Ryu.

## Conflict of interest statement

International patent No. WO2014/086938 describes some of the concepts presented in this manuscript.
